# Metabolic effects of *Foofoo corn* on healthy volunteers: influence of some traditional Cameroonian sauces

**DOI:** 10.1186/s40842-015-0014-4

**Published:** 2015-11-02

**Authors:** Vicky Kamwa, Eugene Sobngwi, Vicky Joceline Ama Moor, Jean Jacques N. Noubiap, Mesmin Dehayem, Crista Arrey-Tabi, Eliane Ngassam, Jean-Louis Nguewa, Leopold Ndemnge Aminde, Eric Djahmeni, Sandrine Ongnessek, Valery Effoe, Barbara Atogho-Tiedeu, Jean-Claude Mbanya

**Affiliations:** 1 National Obesity Center, Yaoundé Central Hospital, Yaoundé, Cameroon; 2 Laboratory for Molecular Medicine and Metabolism, Biotechnology Center, University of Yaoundé I, Yaoundé, Cameroon; 3 Faculty of Medicine and Biomedical Sciences, University of Yaoundé I, Yaoundé, Cameroon; 4 Laboratory of Biochemistry, Yaounde University Teaching Hospital, Yaounde, Cameroon; 5 Department of Medicine, Groote Schuur Hospital and University of Cape Town, Cape Town, South Africa; 6 Medical Diagnostic Center, Yaoundé, Cameroon; 7 Department of Diabetes and Endocrinology, Hôpital Lariboisière, Assistance Publique-Hôpitaux de Paris, University Paris-Diderot Paris-7, Paris, France; 8 Clinical Research Education, Networking and Consultancy, Douala, Cameroon; 9 Internal Medicine Unit, Edéa Regional Hospital, Edéa, Cameroon; 10 Division of Public Health Sciences, Department of Epidemiology and Prevention, Wake Forest School of Medicine, Medical Center Boulevard, Winston‐Salem, NC 27157-1063 USA

**Keywords:** Traditional meal, Glycemic index, Nutrition, Cameroon, sub-Saharan Africa

## Abstract

**Background:**

Little data to guide diet prescription exists about the foods most frequently consumed in Africa. Moreover, the sauce accompanying a meal can significantly alter the metabolic effects of food. Our work was to study the influence of sauces on the metabolic effects of *foofoo corn* (Zea mays), one of the most commonly consumed foods in several countries in sub-Saharan Africa with a wide range of sauces.

**Methods:**

Our study population consisted of ten healthy volunteers (five men, five women), aged from 21 to 28 years, with mean BMI of 23.9 (SD 1.9) kg/m^2^. The study involved seven visits of three hours each, conducted every 2 days, including one devoted to the oral glucose tolerance test (OGTT) and six visits to the consumption of each of 6 meals tested, standardized to 75 g of carbohydrate intake. Blood samples were collected at 0, 15, 30, 60, 90, 120 and 180 min after consumption of meals for blood glucose and triglycerides levels. The glucose area under the curve of each tested meal, was used to calculate its glycemic index, using the OGTT as the reference. The accompanying sauces tested with *foofoo corn* were: *okra sauce* (Abelmoschus esculentus), the so-called *yellow sauce* (Elaeis guinensis), the pistachio sauce (Pistacia vera), the *nkui* (Triumpheta pentandra), *ndolé* (Vernonia amygdalima) and cabbage (Brassica oleracea).

**Results:**

All meals had generally a low glycemic index, with a maximum of 22.59 % for *okra* and cabbage, followed by *ndolè* (20.18 %), the *yellow sauce* (13.10 %), *pistachio sauce* (11.60 %), and *nkui* (5.27 %). There was a difference in the effects of the diets on triglyceride levels only at 180 min (*p* = 0.03).

**Conclusion:**

Whatever the accompanying sauce, *foofoo corn* has a low glycemic index. Some sauces, such as *nkui* give it a very low glycemic index and may be of great interest in diet prescription for patients with various metabolic disorders such as diabetes and obesity.

## Background

Dietary intervention is the cornerstone in the management of type 2 diabetes, obesity and diseases associated with them. Knowing the metabolic effects of foods most consumed in a community is important for the promotion of healthy diets, appropriate and effective management of patients with diabetes in this population. Since the early 1980s, the glycemic index and insulinemic index were suggested as a means of evaluating the effects of food on glucose metabolism [1]. Therefore, studies on the determination of the glycemic index of foods regularly consumed in different countries contributed significant information in diet prescription. Although there are studies referenced in Ghana, South Africa, and Kenya [2–4], data on the composition and effects of traditional foods consumed in Africa are rare. In 2003, Mbanya et al. evaluated the glycemic index and insulinemic index (II) of five meals commonly consumed in Cameroon, including *foofoo corn* + *ndolè*. This proved to be the meal with the lowest glycemic index (34.1 %) [5].

*Foofoo corn* is a staple food that is widely consumed throughout Cameroon and other countries in sub-Saharan Africa. Our study investigated the influence of local sauces on the metabolic effects of *foofoo corn*. Knowledge about the influence of these sauces could help tailor dietary recommendations for this population.

## Methods

### Ethics statement

The study was granted approval by the National Ethical Review Board of the Cameroon Ministry of Public Health (ethical clearance N° 171/CNE/SE/2012). Written informed consent was obtained from all the participants. The study was conducted in accordance with the Helsinki Declaration.

### Subjects and meals

Ten healthy volunteers, non-obese with no history of diabetes, hypertension or gastrointestinal tract surgery, were included in the study after they had given a written informed consent. Their characteristics are summarized in Table 1.

**Table 1:** Characteristics of the study population

Characteristics	Mean	Standard deviation
Age (years)	25	1,0
Body mass index (kg/m^2^)	23,9	1,9
Waist size (cm)		
Male	79	5,0
Female	76	2,0
Systolic blood pressure (mmHg)	114	10,0
Diastolic blood pressure (mmHg)	69	10,0
Estimated GFR (ml/min)	97,3	5,8
Alanine amino-transférase (UI)	28,1	1,7

*GFR* glomerular filtration rate

Six sauces were selected, after we carried out the qualitative phase of the study, which aimed to determine the sauces commonly used accompanying foofoo corn in Cameroon. They were labelled A, B, C, D, E and F. They consisted of *foofoo corn* (*Zea mays*) + *okra* (*Abelmoschus esculentus*) sauce (diet A), *foofoo corn* + *pistachio* (*Pistacia vera*) sauce (diet B), *foofoo corn* + *ndolè* (*Vernonia amygdalima*) (diet C), *foofoo corn* + *yellow sauce* (*Elaeis guinensis*) (diet D), *foofoo corn* + cabbage (*Brassica oleracea*) (diet E), and *foofoo corn* + *nkui* (*Triumpheta pentandra*) (diet F). The portion of each diet served was calculated in order to achieve standardization to 75 g carbohydrate content, 30 g of lipids, 10 g of fiber, and 75 g of proteins, as shown in Annex 1.

### Study protocol

The determination of the glycemic index was done in accordance with the recommendations by Brand-Miller [6]. After selection, the subjects were invited to attend the meal studies on seven visits with 2 days interval between any two visits. They were advised to maintain a minimal physical activity level the day preceding each visit, and attend the exploration after an overnight fast. The visits consisted of a standard oral glucose tolerance test on the first occasion, and the ingestion of diets A, B, C, D, E, and F on the subsequent six visits, respectively. On each visit day, a cannula was placed in a vein of the forearm and kept patent with slow saline infusion (9 g NaCl/l). We did capillary blood glucose, 0 (fasting), 15, 30, 60, 90, 120 and 180 min after the 75 g glucose load (visit 1) or test meal using the Accu-Chek® Compact Plus glucometer (F. Hoffmann-La Roche AG, Basel, Switzerland) which has been shown to have a good accuracy and precision [7], with a very low coefficient of variation (~3 %) [8]. We also collected blood samples in dry tubes for the measurement of triglycerides levels, at those same times. Serum was separated by a 10 min, 3000 tours/min centrifugation at 6 °C, and stored at −80 °C until biochemical explorations. Serum triglycerides (glycerol phosphatase oxidase − phenol4-amino antipyrene peroxidase method) levels were were measured on a spectrophotometer (UV Mini 1240) using Chronolab kits (Chronolab Systems, Barcelona, Spain).

### Statistical analysis

Results were plotted as glucose curves. The glycemic index, defined as the area under the glucose response curve after consumption of a test food, divided by the area under the curve after consumption of a control food containing the same amount of carbohydrate [9, 10], was calculated using 75 g of glucose as the standard; the glycemic index of which was set at 100 %. The change in area under the curve was calculated as the incremental area above the fasting value using the trapezoidal formula.

$$ GI=\frac{area\  under\  the\  curve\  of\  test\  meal}{area\  under\  the\  cuve\  of\  glucose}\times 100 $$

Results are expressed as means ± standard deviation, unless otherwise specified. The Student *t*-test was used for the comparison of each meal to the glucose, and we used ANOVA for the comparison of means of different meals at different times.

## Results

**Fig. 1:**
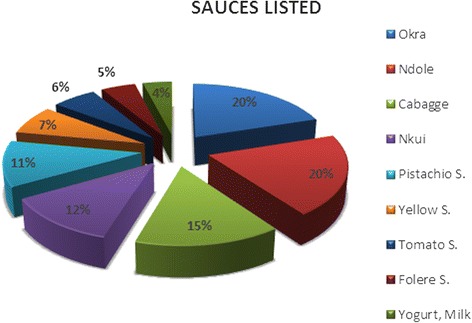
Frequency of sauces most consumed with *foofoo corn*

At the end of our qualitative phase, conducted in 10 restaurants and 20 households, the analysis of the frequency of sauces most found with foofoo corn, showed that the six sauces most consumed were *okra sauce*, *ndolè*, *cabbage*, *nkui*, *pistachio sauce* and the so-called yellow sauce, as shown in Fig. 1.

**Fig. 2:**
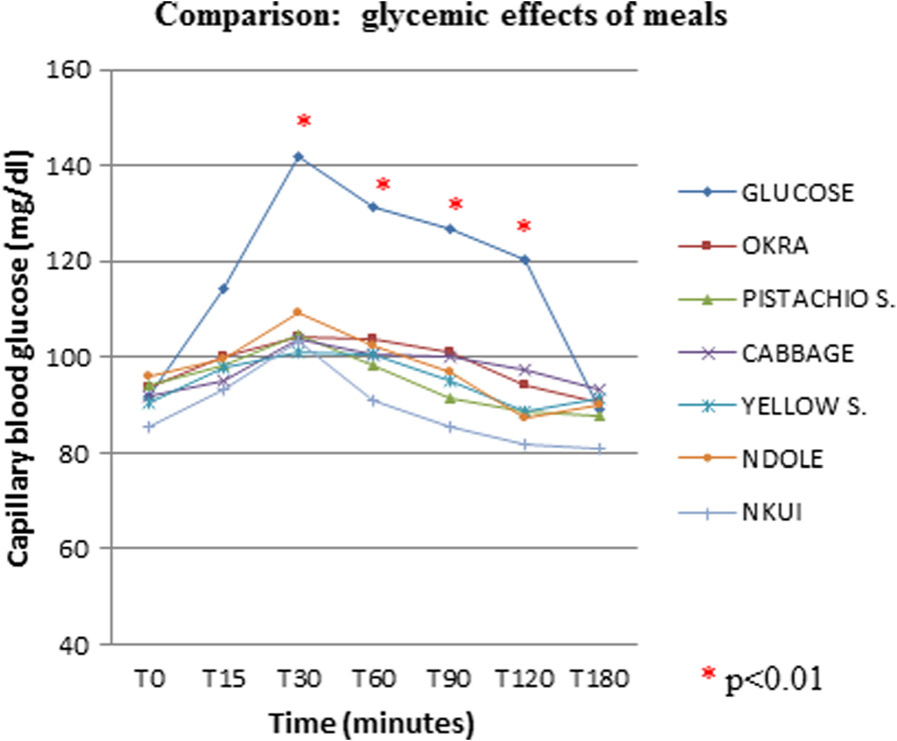
Glycemic effects of meals

For the experimental phase, the capillary blood glucose curves are shown in Fig. 2. The mean blood glucose concentration was similar before ingestion of the test diets on the seven occasions, varying from 0.86 ± 0.10 to 0.91 ± 0.10 g/L. It increased above pre-ingestion values during the first 30 min post-prandial with all the diets (Fig. 2). The highest initial peak was obtained with the 75 g glucose load at 54.3 % increase above basal level, followed by diet C (*foofoo corn* + *ndolè*) (19.6 % increase), and the lowest diet D (*foofoo corn* + *yellow sauce*) (9.8 % increase), with a significant difference between the diets (*P* < 0.01). Blood glucose returned to basal values within 60 min for diets B (*foofoo corn* + *pistachio sauce*) and F (*foofoo corn* + *nkui*), within 120 min for the four others, and within 180 min for the glucose load.

Analysis of variance showed no statistical difference between the glycemic indexes of the selected diets, although it varied from 5.27 % [diet F (*foofoo corn* + *nkui*)] to 22.59 % [diet A (*foofoo corn* + *okra sauce)* and C *(foofoo corn* + *ndolè*)], as shown in Table 2. There was a difference in the effects of the diets on triglyceride levels only at T180 (*p* = 0.03) (Table 3).

**Table 2:** Glycemic index of meals

	Highest glycemia (g/l)	Area under the curve	Glycemic index (%)
Glucose	1,42	49,8	100
Nkui	1,03	2,63	5,27
Pistachio sauce	1,05	5,78	11,60
Yellow sauce	1,01	6,53	13,10
Ndolè	1,10	10,05	20,18
Cabbage	1,04	11,25	22,59
okra sauce	1,04	11,25	22,59

**Table 3:** Serum triglycerides levels following meals

	Time following meal consumption (min)						
	T0	T15	T30	T60	T90	T120	T180
OGTT	1,66	1,77	1,66	1,64	1,53	1,55	1,51
*Okra sauce*	1,66	1,72	1,89	1,62	1,59	1,60	1,59
*Yellow sauce*	1,60	1,74	1,65	1,62	1,59	1,52	1,63
*Pistachio sauce*	1,65	1,78	1,69	1,57	1,64	1,56	1,46
*Nkui*	1,60	1,72	1,70	1,59	1,49	1,47	1,43
*Ndolè*	1,60	1,68	1,73	1,57	1,59	1,50	1,58
*Cabbage*	1,66	1,72	1,81	1,64	1,63	1,56	1,53
ANOVA(F)	0,33	0,28	0,79	0,46	2,13	0,52	2,56
P-value	0,93	0,94	0,58	0,84	0,06	0,79	0,03

*OGTT* oral glucose tolerance test

## Discussion

This study provides information on the influence of six sauces on the metabolic effects of the most widely consumed staple food in Cameroon, foofoo corn, through the determination of the glycemic index of the meal combinations, for the glycemic effects, and the determination of triglycerides for metabolic effects, in healthy subjects.

The sauces used in descending order were: *okra sauce* (20 %), *ndolè* (20 %), cabbage (14.7 %), *nkui* (12 %), *pistachio sauce* (11.3 %) and *yellow sauce* (7.3 %). The glycemic indexes ranged from 5.27 % [diet F (*foofoo corn* + *nkui*)] to 22.59 % (% [diet A (*foofoo corn* + *gombo)* and C *(foofoo corn* + *ndolè*)] with a statistically significant difference between all meals, from T_30_ to T_120_ (*p* < 0.01). In contrast, for the determination of the effects of the diets on triglycerides, the difference was at T_180_ (*p* = 0.03), showing that triglyceride levels were not altered acutely. The highest values were generally found at T_15_. These results could be explained by the presence of chylomicrons normally high in the early postprandial period. The significant lower levels of serum triglycerides found at T_180_ (latest postprandial period) is probably due to the breakdown of triglycerides by lipoprotein lipase which leads to reduction in their serum levels.

There are limitations when the coefficient of variation of the glucometer used is not validated. But in our study, we used a glucometer that has a very low coefficient of variation [7, 8]. Moreover, the glycemic index can be determined relative to white bread taken as the reference food, but also with glucose, as is the case in this study.

The sample size may seem small, but it is comparable to the recommendations of WHO and FAO, 1998, and other similar studies, like that of Brakohiapa and coworkers, who studied with ten healthy young adult men, the glycemic response of five commonly consumed foods in Ghana [2] or Mbanya et al. who determined the metabolic and hormonal effects of five most commonly consumed foods in Cameroon [5] in ten healthy young adults. However, studies with larger sample size are needed to draw definitive inferences.

The subjects included in this study had no relevant medical history and physical examination was unremarkable. Additionnally, they had no hyperglycemia in the second hour post load, proving their healthy state. The same criteria of selection of healthy subjects were used by Mbanya et al., Brand-Miller at al. and Jenkins et al. [1, 5, 11] .

Possible changes that could have affected our results include variations induced, either by the way of food preparation, which differs between households and restaurants, either by the nature or the different degrees of maturity of the needed ingredients for the preparation of the meal, since it is finally the combined effect of all components that influence the value of the glycemic index of a food [12]. In a bid to limit such bias in our study, we used a standardized procedure for the cooking of all these meals. The recipes were taken from a recipes book adopted in the country, entitled “Le Cameroun se met à table”.

In other countries, such as in Mexico, according to a study of Frati-Munari et al., the glycemic index of foods tested, ranged from 10 % ± 17 % for the cactus, to 54 % ± 15 % for brown beans, after a repeated test 14 to 18 times for the same food [13]. In India, Urooj et al. determined the glycemic index of foods made up of six cereals commonly eaten and the values ranged from 44 to 69 % in healthy subjects [14]. With values ranging from 5.27 to 22.59 %, the meals tested in our study are likely to have lower glycemic indices than those previously tested as seen in a study by Mbanya et al. in Cameroon, in which the results of the five foods tested showed the lowest glycemic index for foofoo corn + ndolè (34.1 %) [5]. In this study, the latter has its glycemic index lowered up to 20.18 %. This is probably due to the addition of meat while cooking and to the presence of fat. Indeed, glucose responses of a food eaten alone or in combination with other foods differ. Adding fat or protein to a carbohydrate meal also enhances insulin secretion even though the plasma glucose response actually decreases [15–18]. Moreover, all three primary macronutrients (carbohydrate, fat and protein) stimulate the release of several gut peptides, but to different degrees, and influence glucose effect. Protein and fat are particularly efficacious in stimulating gut peptide release despite a small direct glucose effect [15–18]. Another explanation could be the difference in the mode of preparation of these meals [12], with variation in the respective recipes. The *nkui* is a traditional sauce prepared with many ingredients, roots and barks. Its glycemic index, determined at 5.27 % ranks it among the foods with extremely low glycemic index [19]. During its preparation, no considerable amount of protein was added that would justify such a result. Therefore, it may have an ingredient, or the combination of several ingredients, or an intrinsic glucoregulatory effect of the plant itself, which may be responsible for the hypoglycemic effect. Another explanation is its composition - rich in fiber and minerals, with little or no fat and carbohydrates, probably accounting for blood glucose reduction, and therefore, the glycemic index. Finally, glycemic index is dependent on the history of the processing, storing, ripening, cutting, and cooking of the food [20].

There is great controversy about the utility of using glycemic index in the management diabetes and certainly obesity. Although glycemic index presents some drawbacks, it may be useful in dietary prescription [21] as some studies have shown the efficiency of the consumption of low glycemic index meals in the management of diabetes, obesity and related diseases [11, 22, 23]. Indeed, there is evidence that low glycemic index diets are effective in improving glucose metabolism and insulin sensitivity as well as various markers of cardiovascular risk in people with diabetes and obesity and can be considered in the overall strategy of diabetes management [24–27]. The results of this study only lengthen the list of foods that could be potentially recommended for Cameroonian patients with metabolic disorders, to vary their diet and avoid stress related to culinary cultural differences. Further studies are needed to confirm the potential benefit of these low glycemic index diets for dietary interventions.

## Conclusion

This study suggests that whatever the sauce that accompanies *foofoo corn*, it could have a substantial role in diet therapy, without fear of significant overall effect on carbohydrate and lipid metabolism. Some sauces such as *nkui* give it a very low glycemic index and may be of great interest in diet prescription for patients with various metabolic disorders such as diabetes.
